# Supplementary material for: Gas Chromatographic Fingerprint Analysis for the Comparison of Seized Cannabis Samples
Source: Molecules. 2021 Nov 2;26(21):6643. doi: 10.3390/molecules26216643 (PMC8587667; doi:10.3390/molecules26216643)
Supplement: Supplementary file 1 [file molecules-26-06643-s001.zip › molecules-1423644-supplementary.pdf]

molecules

MDPI

Supplementary Materials

Gas Chromatographic Fingerprint Analysis for the Comparison of Seized Cannabis Samples

Amorn Slosse [1,2], Filip Van Durme [1], Nele Samyn [1], Debby Mangelings [2] and Yvan Vander Heyden [2,*]

[1] Drugs and Toxicology Department, National Institute for Criminalistics and Criminology (NICC), Vilvoordsesteenweg 100, B-1120 Brussels, Belgium; amorn.slosse@just.fgov.be (A.S.); Filip.VanDurme@just.fgov.be (F.V.D.); Nele.samyn@just.fgov.be (N.S.)
[2] Department of Analytical Chemistry, Applied Chemometrics and Molecular Modelling, Vrije Universiteit Brussel (VUB), Laarbeeklaan 103, B-1090 Brussels, Belgium; Debby.Mangelings@vub.be
* Correspondence: yvanvdh@vub.be

Abstract: *Cannabis sativa L.* is widely applied as recreational illegal drugs. Illicit cannabis profiling, comparing seized samples, is challenging because of the natural Cannabis heterogeneity. The aim of this study is to use GC-FID and GC-MS herbal fingerprints for intra (within)- and inter (between)-location variabilities evaluation. This study focuses on finding an acceptable threshold to link seized samples. Through Pearson correlation-coefficient calculations between intra-location samples, 'linked' thresholds were derived using 95% and 99% confidence limits. False negative (FN) and false positive (FP) error rate calculations, aiming at getting the lowest possible FP value, were performed for different data pretreatments. Fingerprint-alignment parameters were optimized using Automated Correlation Optimized Warping (ACOW) or Design of Experiments (DoE), which resulted in similar results. Hence, ACOW data, as reference, showed 54% and 65% FP values (95/99% confidence). Additional fourth root normalization pretreatment provided the best results for both the GC-FID and GC-MS data sets. For GC-FID, which showed the best improved FP error rate, 54/65% FP for the reference data decreased to 24 and 32% after fourth root transformation. Cross validation showed similar FP values as the entire calibration set, indicating the representativeness of the thresholds. A noteworthy improvement in discrimination between seized Cannabis samples could be concluded.

Keywords: Chromatographic fingerprint; alignment optimization; design of experiments; data preprocessing; comparison intra- and inter-location samples

Citation: Slosse, A.; Van Durme, F.; Samyn, N.; Mangelings, D.; Vander Heyden, Y. Gas chromatographic fingerprint analysis for the comparison of seized cannabis samples *Molecules* **2021**, *26*, 6643. https://doi.org/10.3390/molecules26216643

Academic Editor: Michal Daszykowski, Joaquim Jaumot, Hadi Parastar

Received: 30 September 2021
Accepted: 26 October 2021
Published: 2 November 2021

Publisher's Note: MDPI stays neutral with regard to jurisdictional claims in published maps and institutional affiliations.

Copyright: © 2021 by the authors. Licensee MDPI, Basel, Switzerland. This article is an open access article distributed under the terms and conditions of the Creative Commons Attribution (CC BY) license (http://creativecommons.org/licenses/by/4.0/).

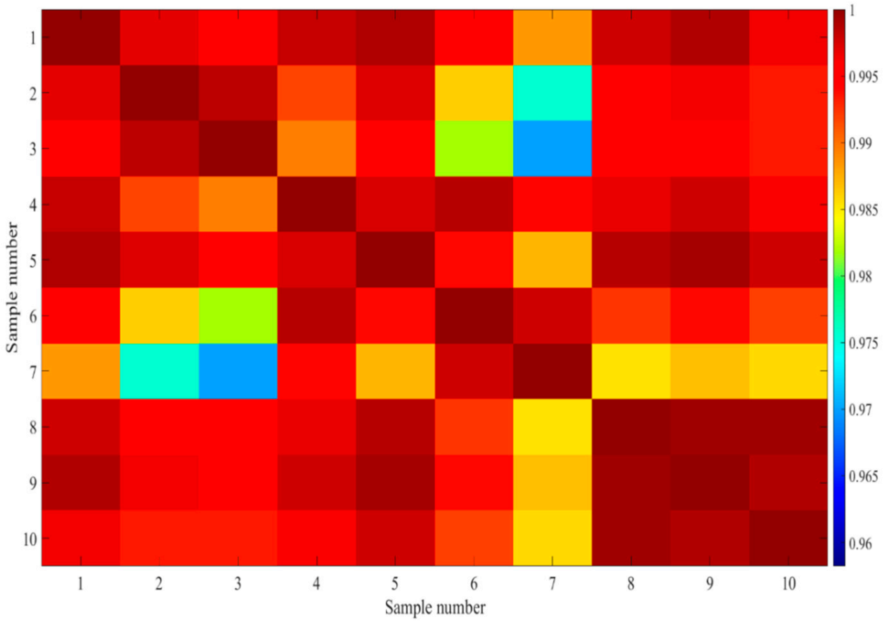

Figure S1. Intra-correlation coefficients colour map from the 8th cultivation site. GC-FID data were used.

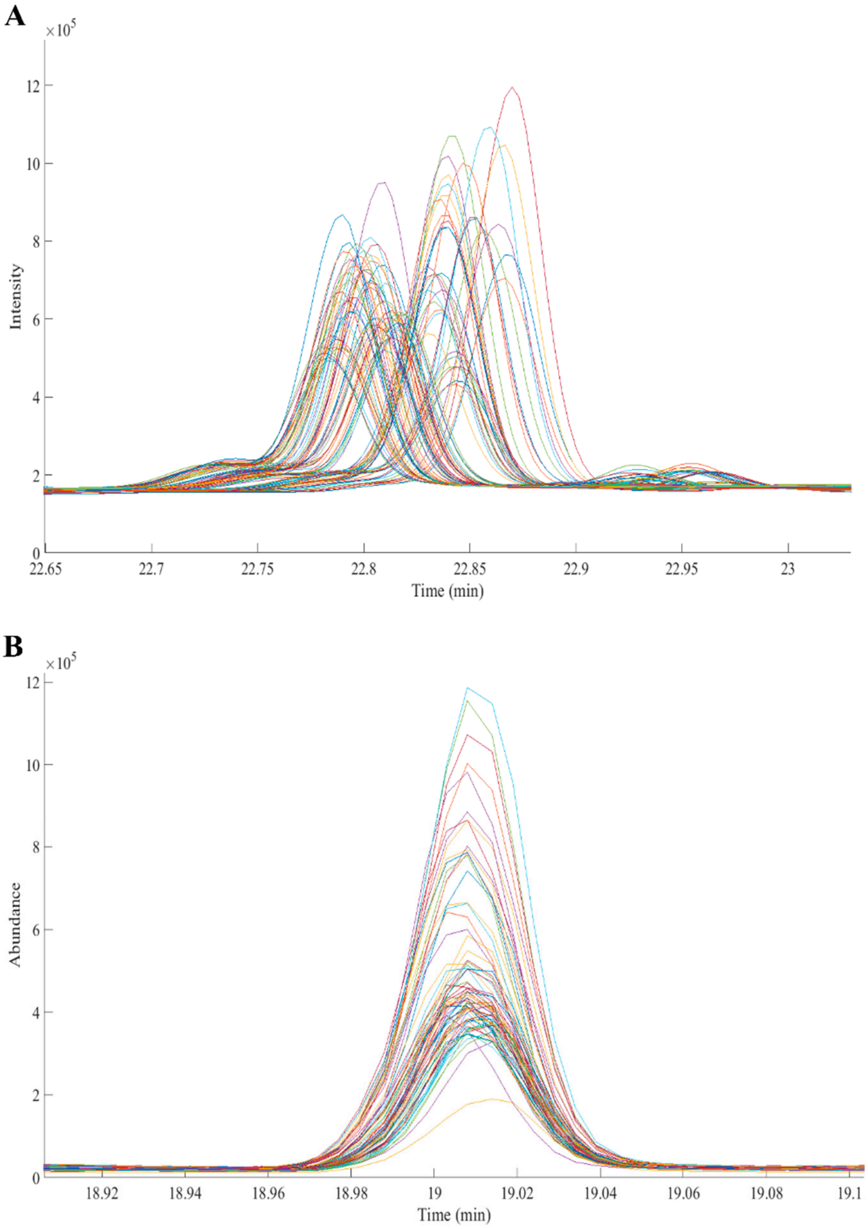

Figure S2. A detail plot of one peak for (A) GC-FID raw data, and (B) GC-MS TIC raw data for the 10 different cultivation sites.

Table S1. Summary of the FN and FP values of GC-FID Cannabis fingerprints for the ACOW and DoE approach. The effect of multiple alignments are also shown. The best FP error rates are denoted in bold.

	**95% CL**		**99% CL**	
	FN (%)	FP (%)	FN (%)	FP (%)
**Raw Data**	6	34	3	39
**Range SL 15-100 & SS 1-10**				
1 x ACOW	6	57	4	65
2 x ACOW	6	**47**	4	**55**
3 x ACOW	5	49	4	58
1 x DoE	5	**47**	3	**55**
2 x DoE	4	58	3	65
3 x DoE	6	50	4	59

**Range SL 25-200 & SS 1-10**				
1 x ACOW	6	58	4	63
2 x ACOW	5	54	4	62
3 x ACOW	5	66	2	75
1 x DoE	6	58	4	60
2 x DoE	5	57	3	62
3 x DoE	4	**49**	3	**55**

Table S2. Representation of the alignment optimization on the GC-MS raw data. The warping effect is expressed as the obtained FN and FP error rates.

	95% CL		99% CL	
	FN (%)	FP (%)	FN (%)	FP (%)
**Raw Data**	4	53	3	58
**Range SL 15-100 & SS 1-10**				
1 x ACOW	7	53	3	58
1 x DoE	7	53	3	58
**Range SL 25-200 & SS 1-10**				
1 x ACOW	6	54	2	57
1 x DoE	6	54	3	57

Table S3. The obtained total % FN and FP for all pretreatment techniques after aligning the GC-FID raw data twice using ACOW.

**Pre-treatment method**	**95% CL**		**99% CL**	
	**FN (%)**	**FP (%)**	**FN (%)**	**FP (%)**
**Aligned data (2xACOW)**	6	47	3	55
**Column centering (CC)**	9	55	6	63
**Normalization + CC (N+CC)**	7	54	4	67
**Standard normal variate + CC (SNV+CC)**	7	52	4	66
**Square root normalization**	7	29	3	38
**Fourth root normalization**	7	24	4	32
**Auto-scaling**	6	19	4	27

Table S4. Total % FN and FP for all pretreatment techniques after removal of THC of the aligned GC-FID data matrix.

**Pre-treatment method**	**95% CL**		**99% CL**	
	**FN (%)**	**FP (%)**	**FN (%)**	**FP (%)**
**Aligned data (1xACOW)**	6	57	4	65
**Column centering (CC)**	7	31	3	42
**Normalization + CC (N+CC)**	4	29	3	40
**Standard normal variate + CC (SNV+CC)**	7	29	3	38
**Square root normalization**	5	30	4	40
**Fourth root normalization**	5	28	4	37
**Auto-scaling**	6	19	4	27
